# Palindromic Nucleotide Analysis in Human T Cell Receptor Rearrangements

**DOI:** 10.1371/journal.pone.0052250

**Published:** 2012-12-21

**Authors:** Santosh K. Srivastava, Harlan S. Robins

**Affiliations:** 1 IBM Research Lab, New Delhi, India; 2 Programs in Computational Biology, Fred Hutchinson Cancer Research Center, Seattle, Washington, United States of America; National Institute on Aging, United States of America

## Abstract

Diversity of T cell receptor (TCR) genes is primarily generated by nucleotide insertions upon rearrangement from their germ line-encoded V, D and J segments. Nucleotide insertions at V-D and D-J junctions are random, but some small subsets of these insertions are exceptional, in that one to three base pairs inversely repeat the sequence of the germline DNA. These short complementary palindromic sequences are called P nucleotides. We apply the ImmunoSeq deep-sequencing assay to the third complementarity determining region (CDR3) of the β chain of T cell receptors, and use the resulting data to study P nucleotides in the repertoire of naïve and memory CD8^+^ and CD4^+^ T cells. We estimate P nucleotide distributions in a cross section of healthy adults and different T cell subtypes. We show that P nucleotide frequency in all T cell subtypes ranges from 1% to 2%, and that the distribution is highly biased with respect to the coding end of the gene segment. Classification of observed palindromic sequences into P nucleotides using a maximum conditional probability model shows that single base P nucleotides are very rare in VDJ recombination; P nucleotides are primarily two bases long. To explore the role of P nucleotides in thymic selection, we compare P nucleotides in productive and non-productive sequences of CD8^+^ naïve T cells. The naïve CD8^+^ T cell clones with P nucleotides are more highly expanded.

## Introduction

Surveillance of intracellular proteins are primarily mediated by T cells which recognize processed antigens presented by major histocompatibility complex (MHC) molecules on the cell surface. CD8^+^ T cells recognize endogenous proteins processed and transported through the endosomal pathway and presented by class I MHC molecules, while CD4^+^ T cells recognize exogenous proteins presented by class II MHC molecules. The specificity and affinity of T cell recognition is largely contained in α and β chains of the T cell receptor (TCR). TCR sequence diversity resides primarily in the complementarity determining region 3 (CDR3) loops of α and β chains, which bind to the peptide antigen, conveying specificity. The nucleotide sequence that encodes the CDR3 loops are generated by V(D)J recombination: variable (V_β_), diversity (D_β_) and joining (J_β_) genes in the genome are rearranged to form a β chain, while V_α_ and J_α_ genes rearrange to form an α chain.

The large TCR repertoire arises as a consequence of combinatorial and junctional diversity. Existence of multiple V_β_, D_β_ and J_β_ genes in the genome and the number of ways V_β_, D_β_ and J_β_ genes can recombine give rise to combinatorial diversity. Junctional diversity is generated by deletion of nucleotides adjacent to the recombination signal sequences (RSSs) of the V_β_, D_β_ and J_β_ gene segments and non-templated insertion of random nucleotides (N nucleotides). N nucleotides which increase the diversity by exponential order are inserted by the DNA polymerase terminal deoxynucleotidyl transferase (TdT) at the V_β_−D_β_ and D_β_−J_β_ junctions in a template independent manner for the TCRβ chain. In this manuscript we will denote the nucleotide insertion at D_β_−J_β_ and V_β_−D_β_ junctions as N1 and N2 regions, respectively.

To express a complete TCR in a developing T cell, an initial DNA break is performed by the recombination activating gene RAG1-RAG2 complex, which binds to the RSS and introduces a DNA double-strand break at the precise border of the coding end and the RSS. The ends bearing the coding sequence are sealed covalently and form transient hairpin loops, which are then cut open and digested to varying extent by an exonuclease. The position of the cut(s) at various points along the hairpins by the Artemis enzyme phosphorylated by a DNA-dependent protein kinase (DNA-PK) plays a key role in the formation of sequence variability in the final joint of the TCR loop. Usually single strand cuts on both strands of the hairpin result in deletion of a few nucleotides at the coding ends. However, for a subset of rearrangements, an asymmetric cut on only one strand of the hairpin transfers nucleotides from one strand to the other and results in a 3′ or 5′ protruding single strand which is complementary with respect to the end of the coding sequence. These short complementary palindromic sequences are called P nucleotides [1], [2], [3], [4], the primary focus of this manuscript. Here we will denote the observed and true complementary sequence as palindrome and P nucleotide respectively.

P nucleotides were first discovered in 1993 by [5] Thompson et al. However, little information is available on the in vivo role of P nucleotides in the TCR. P nucleotides are usually short (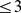 nts), but their location in the CDR3 regions of TCRs suggests that they can significantly influence the cellular adaptive immune response. Since the TCRβ CDR3 is of fixed length and has a primary role in antigen recognition, any small perturbation in the nucleotide sequence encoding the TCR could affect the T cell drastically. Thus P nucleotides in the TCR could affect both positive and negative selection in the thymus and can help determine antigen recognition in the periphery. If we do not consider P nucleotide in TCR receptor analysis, we would overestimate the number of nucleotides inserted by the TdT and this in turns overestimate the TCR sequence diversity. Furthermore, understanding P nucleotides is a prerequisite for studying the pattern of nucleotide distributions contributed by the TdT enzyme.

Our knowledge of P nucleotides is incomplete due to limited amount of TCR sequence data available. Applying the ImmunoSeq deep-sequencing assay to TCRs, we sequence millions of TCR sequences in parallel from a single sample, creating a large data set of TCRβ CDR3 sequences, which we utilize to study P nucleotides in a unified and coherent manner [6], [7]. We are able to estimate P nucleotide distributions in individuals and then compare across a cohort of healthy people and different T cell subtypes.

In this study, we analyze P nucleotides in the repertoire of TCRβ CDR3 sequences from the genome of naïve and memory CD8^+^ and CD4^+^ T cells that were obtained from the peripheral blood of seven healthy people. We build a linear model to characterize P nucleotide probabilities, and apply this model to each observed palindromic sequence to assign a probability that the sequence is a P nucleotide or a random insertion by TdT. This probability model can be directly used as part of an algorithm to decompose CDR3 junctions into their constituent parts. We elucidate the properties of P nucleotides in different subsets of T cells and across different donors using this model. We show that the distribution of P nucleotides are highly biased with respect to gene segment usages and its coding end and the majority of observed palindromes are not actually P nucleotides. We also show that P nucleotide distribution in productive sequences is different from non-productive sequences, and longer P nucleotides at the specific coding end is biased towards the high average copy number in CD8^+^ naïve T cell. Additionally, we show that junctions with P nucleotide sequences have fewer non-template insertions and induce a reading frame bias in the β chain of the TCR.

## Results

We applied a high-throughput sequencing approach [6] to estimate the P nucleotide distribution in the naïve and memory compartments of CD8^+^ and CD4^+^ T cells obtained from the peripheral blood of healthy adults. Genomic DNA was purified from both the naïve (CD45RO^−^, CD45RA^hi^, CD62L^hi^) and memory (CD45RO^+^, CD45RA^int/neg^) CD4^+^ and CD8^+^ T cells (**Table S1, Table S2**). More than 5 million TCRβ CDR3 sequence reads were generated from ∼1 million template genomes in each of the naïve and memory samples. A mean of 400,000 CD8^+^ naïve, 60,000 CD8^+^ memory, 340,000 CD4^+^ naïve, and 170,000 CD4^+^ memory unique CDR3 nucleotide sequences were observed (**Table 1**).

**Table 1:** Summary of TCRβ CDR3 sequence data.

T cell population	Mean		Coding end	Number of observed palindromes of length						
	Reads	Unique		1	2	3	4	5	6	7
CD8^+^ Naive	5,569,034	398,084	3′ V	7220	3414	944	248	81	12	2
			5′ D	8668	10647	3910	635	165	14	3
			3′ D	7404	2659	485	96	30	8	1
			5′ J	4298	4867	1229	277	73	11	1
CD8^+^ Memory	7,087,197	60,651	3′ V	741	337	78	23	7	1	0
			5′ D	883	953	295	45	12	2	1
			3′ D	1267	346	60	10	2	1	0
			5′ J	460	400	83	23	7	1	0
CD4^+^ Naive	8,121,324	340,062	3′ V	5970	3423	758	198	57	11	1
			5′ D	7337	9660	3392	555	162	12	3
			3′ D	7551	2845	515	109	30	5	1
			5′ J	4121	4273	1054	254	78	14	0
CD4^+^ Memory	8,339,225	172,919	3′ V	2836	1536	345	90	25	6	0
			5′ D	3548	4497	1555	240	71	6	1
			3′ D	4036	1459	260	43	14	2	0
			5′ J	2000	2008	491	120	34	4	1

The mean number of in-frame, read-through TCRβ CDR3 nucleotide sequence reads obtained from the CD8**^+^**CD45RO**^−^**CD45RA**^hi^**CD62L**^+^** (naïve), CD8**^+^**CD45RO**^+^**CD45RA**^low^** (memory), CD4**^+^**CD45RO**^−^**CD45RA**^hi^**CD62L**^+^** (naïve) and CD4**^+^**CD45RO**^+^**CD45RA**^low^** (memory) T-cell samples. The last seven columns show the average counts of palindromes of length one to seven observed in the data set at four different non-recessed coding ends. Palindromic sequence longer than 7 nucleotides long is not observed in the data set.

### Method Overview

We briefly review the linear probability model for characterizing P nucleotide distribution, referring to the Materials and Methods for a full and technical description. The probabilistic model is designed to address the problem that palindromic nucleotides can occur from two different sources, true P nucleotides and random insertions from TdT that happen to be palindromic by chance. The linear probability model uses the palindromic and TdT (N nucleotides) data of T cell receptors of ethnically diverse individuals and aims to estimate the P nucleotide probabilities of different length. Unlike the method described in the earlier works [3], [4] in which P nucleotides were scored as a P-value using a binomial distribution, the method described here is non-parametric, and does not rely on data belonging to any particular distribution. This approach being non-parametric, based on fewer assumptions, is more robust and has a wider applicability. In linear probability models a linear system is described at each coding end of the given gene segment. It takes as inputs the palindrome length frequencies in the cell population and the TdT insertion probabilities at each position, and output the probability of P nucleotides of varying lengths. The empirical frequencies of palindrome lengths are calculated directly from the data set. The estimation of TdT insertion probability is described below using an assumption that TdT adds nucleotides independently.

At each of the four non-recessed coding ends of the CDR3β chain of T cell receptors either there is no P nucleotide or P nucleotide due to hairpin structure. If P nucleotides arise, they are usually followed by random N nucleotide insertions by the TdT enzyme. Given the rearranged CDR3β chain of T cell receptor, there is no well-defined boundary between the P and N nucleotides to decompose them into disjoint sequences without error. Observed palindrome sequences arise purely by true P nucleotides due to hairpin or purely by N nucleotides due to the TdT enzyme, or by a joint combination of both. We establish a linear system to estimate the P nucleotide probabilities of different length with the assumption that each equation of the system quantifies the observed palindrome length frequency as a mixture combination of P nucleotide and N nucleotide probability distributions.

Modeling palindromic sequences up to *m* bases gives rise to an *m* by *m* linear system, where the unknowns are the probabilities of P nucleotide from 1 to *m* base long. The observed frequency f_n_ that the first *n* nucleotides (*n*≤*m*) are self-complementary or palindromic is equal to the sum of the probabilities that these nucleotides are independently added by the TdT or P nucleotides are followed by N nucleotides added by the TdT enzyme or that an observed palindromic sequence has at least an *n* base P nucleotides. Thus in each equation of the linear system the palindrome length frequency f_n_ can be expressed mathematically as a sum of three terms. These three terms represent the three different biological processes that give rise to palindromes in T cell receptors. The first term represents all *n* nucleotides are independently added by the TdT enzyme, and thus this term encodes the well-known TdT bias for inserting particular nucleotides. The second term represents the first *i* base is a P nucleotide, where *i*≤*n−1*, and the remaining *n−i* bases are N insertions; and the third term represents that there is an at least *n* base P nucleotides. In general, the vector of palindrome length frequency [f_1_, f_2_,… f_M_]^T^ can be written as a sum of the TdT probability vector, TdT bias, and a matrix *A* times the vector of unknown P nucleotide probability [p_1,_ p_2,_… p_M_]^T^. The matrix *A* encodes the information content of the biological steps that gives rise to P nucleotides. The P nucleotide probability vector is solved by inverting the matrix, *A*, and multiplying it by the difference of palindrome length frequency vector [f_1_, f_2_,… f_M_]^T^ and the TdT probability vector.

**Figure 1:**
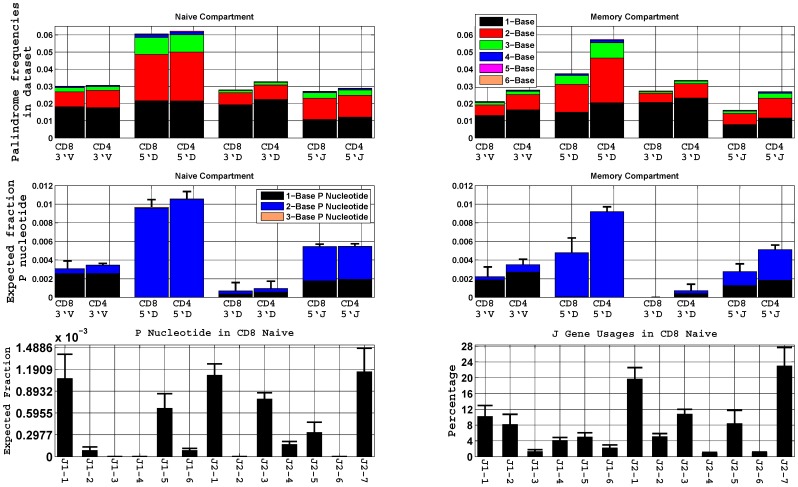
Distributions of P nucleotides. (**A**) Histogram of the average palindrome length frequencies at four coding ends of the V(D)J rearrangement in the naïve and memory CD8^+^ T cells of seven healthy donors and the naïve and memory CD4^+^ T cell of six donors. Each entry is normalized by its total number of unique sequences. (**B**) Mean expected fraction of P nucleotides at the four coding ends. P nucleotides probabilities are calculated using a linear probability model. The expected number of P nucleotides is calculated using these probablities in each donor and then normalized by its total number of unique sequences. The mean height is shown in the figure. P nucleotides mostly occur at the 5′ end of D_β_ followed by the 5′ end of J_β_ and 3′ end of V_β_. Different colors represent expected fraction of one, two and three base P nucleotides. One base P-nucleotides are more common at the 3′ and the 5′ of V_β_ and J_β_, while two base P nucleotides are most common at the 5′ of D_β_. The error bars indicate one standard deviation of the sum of the total P nucleotides. (**C**) Show the comparison of P nucleotide distribution at the 5′J_β_ with J_β_ gene segment usage in TCRβ CDR3 sequences observed in CD8^+^ naïve T cells. The error bars indicate the standard deviation of seven donors.

In this manuscript we restrict P nucleotide analysis of palindromic sequences up to *M* = 3 bases because of the following two reasons. First, many of the palindromic sequences in the data set fall in the range of 1 to 3 nucleotides long that gives rise to average palindrome length of 1.62±0.02. The **Table 1** shows the actual counts of the palindrome length in the data set of each T cell compartment, and the histogram of the palindrome length frequencies is given in **Figure 1A**. Second, though we see longer palindromes greater than equal to 4 nucleotides long in our data set, their frequencies fall down drastically, more than a power of 4. Moreover, these longer palindromes only account for less than 2–3% of the total palindromes.

In order to solve for P nucleotide probability the linear system requires the knowledge of TdT probabilities. To estimate the TdT insertion probabilities based on the assumption that N nucleotides are added independently, we take the middle regions of N2 and N1 segments and calculate the mono nucleotide frequencies. These middle regions must be added by TdT insertions. The regions towards the either end of N1 or N2 segments have potential to be biased due to P nucleotide additions and other unknown factors, thus these regions do not account for a true contribution of TdT insertions. For instance, to estimate the TdT probabilities towards the 5′ end of the D_β_ gene segment, N2 segments of seven nucleotides long with no deletion at the 5′ end of the D_β_ gene segment are used and nucleotide frequencies in their middle regions (3^rd^, 4^th^ and 5^th^ bases from the coding end) are calculated. We observed that the nucleotide frequencies at the 3^rd^, 4^th^ and 5^th^ bases from the 5′ of D_β_ segment do no vary significantly and are consistent among all donors (data not shown). We took the nucleotide distribution at the 3^rd^ base as an estimate of the TdT probabilities. Similarly the TdT probabilities at the 3′ of V_β_ and D_β_ and the 5′ J_β_ of coding ends are estimated.

Mean estimated TdT probabilities for T cells are given in **Table S3**. The TdT has a strong bias for certain nucleotides like G/C. In addition we also found that nucleotides inserted by the TdT are biased with respect to the coding end. For example, nucleotides towards the 3′V_β_ coding end is T rich, while nucleotides towards the 5′ and the 3′ of the D_β_ coding ends are G and C rich respectively. We substitute the coding end specific TdT probabilities and empirical frequencies of palindromes into the linear system and solve for the P nucleotide probabilities of different length.

### Distribution of P Nucleotide

We applied the above linear model to each coding end of the V_β_, D_β_ and J_β_ gene segments of TCRs and solved for P nucleotide probabilities. We used these probabilities to calculate the expected number of one, two and three bases P nucleotides in each data set and then normalized by dividing by the total number of unique sequence in the data set. Normalizing this way allows comparison of the results between donors and across T cells subtypes. Several features of P nucleotide distributions were established and characterized.

P nucleotides were observed in both naïve and memory compartments of the CD8^+^ and CD4^+^ T cells (**Figure 1B**). Total P nucleotide percentages in the CD8^+^ and CD4^+^ naive compartments were 1.8% and 1.9% respectively while the corresponding percentages in the CD8^+^ and CD4^+^ memory compartments were 0.9% and 1.7% respectively.

Some coding ends have been found to display a high proportion of P nucleotides while the others have few or none (**Figure 1B**). P nucleotides are mainly seen at the 5′ of D_β_ gene segments, they range from 1% of data set in the naïve T cells to 0.5–0.9% in the memory T cells. On the other hand, we observed very few P nucleotides at the 3′ of D_β_ despite the fact that there were comparable numbers of sequences with no deletion at this coding end (**Table S1 and Table S2**). We have learned from our previous work that the D_β_1 and D_β_2 gene segments are utilized almost uniformly [6] in TCRs from both the naïve and memory T cells. In contrast the P nucleotide distribution was strongly biased with more P nucleotide seen at the D_β_1 than at the D_β_2 gene segment.

We found that 0.5% of each data set contained P nucleotides at the J_β_ coding ends, with the CD4^+^ and CD8^+^ naïve T cells showing a similar distribution while in the memory compartment the CD4^+^ is two fold higher than the CD8^+^. We showed in our previous work that the J_β_ gene usages observed in the 4 different cell types were relatively constant within a given donor [7]. We compared the P nucleotide frequency at J_β_ coding ends with the J_β_ gene usages in CD8^+^ naïve T cells. We found that P nucleotides are mainly seen in the members of the J_β_2 family and correlate with their usages, while in the J_β_1 family P nucleotides are mainly seen at J_β_1-1 and J_β_1-5 and do not necessary correlate with their usages (**Figure 1B**). The coding end 3′V_β_ contributed few (0.3%) P nucleotides as compared to the 5′D_β_ and 5′J_β_, and their distributions were similar in both compartments of the CD8^+^ and CD4^+^ T cells. Most of the P nucleotide contribution at 3′V_β_ came from the gene segments of the V_β_4 family and the V_β_20 gene segment in the naïve and memory populations of the CD8^+^ and CD4^+^ T cells. P nucleotide additions have a different average length depending upon the particular coding end involved. The majority of P nucleotides consist of one nucleotide at 3′V_β_, two nucleotides at the 5′D_β_, and both one and two nucleotides at the 3′J_β_ (**Figure 1B**). Our model also identified some three base P nucleotides at the 5′D_β_, though their expected frequencies were 150 and 400 fold lower in the naïve and memory compartment, respectively than the two base P nucleotides. Most observed three base or longer palindromes are two base P nucleotides that appear to look longer because of palindromic N nucleotide additions added by the TdT enzyme.

### Assigning P Nucleotide Class

Several algorithms classify all *n* base palindromes as *n* base P nucleotide additions, which overestimate P nucleotide distribution in T cell repertoire [8]. For each coding end and the gene segment we calculated the conditional probability of P nucleotide length given an observation of a palindrome in the naïve and memory compartment of the CD8^+^ and CD4^+^ T cells. Technical description of an algorithm for assigning P nucleotide using the conditional probability model is described in Materials and Methods. As it was described and reasoned in the above section, Method Overview, calculation is done for one to three bases long palindromes and the classification is performed according to the maximum conditional probability. We found that conditional probabilities behave very similarly in all populations of T cells. The average result of all the donors of all cell types is shown in the table (**Table 2** **)** and the average result for each cell type is not shown but available on request.

**Table 2:** Conditional probability of P nucleotide.

Gene	Number of observed palindrome of length			1-basepalindrome	2-base palindrome			3-base palindrome			
	1	2	3	P_1_	**P_0_**	P_1_	P_2_	P_0_	P_1_	P_2_	P_3_
TRBV2	10	5	6	0.18	**0.57**	0.13	0.30	**0.42**	0.09	0.19	0.30
TRBV3-1	83	29	13	0.29	**0.57**	0.23	0.20	**0.48**	0.21	0.20	0.11
TRBV4-1	344	159	43	0.27	**0.44**	0.17	0.39	**0.43**	0.17	0.39	0.01
TRBV4-2	248	97	39	0.24	**0.51**	0.17	0.32	**0.49**	0.17	0.31	0.03
TRBV4-3	420	166	63	0.30	**0.57**	0.23	0.20	**0.55**	0.21	0.20	0.04
TRBV7-2	90	22	14	0.25	**0.70**	0.24	0.06	**0.58**	0.19	0.06	0.17
TRBV7-3	62	11	6	0.19	**0.79**	0.19	0.02	**0.75**	0.18	0.02	0.05
TRBV7-6	57	18	6	0.36	**0.53**	0.32	0.15	**0.52**	0.32	0.15	0.01
TRBV7-8	57	16	9	0.37	**0.53**	0.32	0.15	**0.45**	0.28	0.15	0.12
TRBV7-9	57	12	7	0.20	**0.75**	0.19	0.06	**0.65**	0.16	0.06	0.13
TRBV9	41	23	7	0	**1.00**	0	0	**1.00**	0	0	0
TRBV10-1	22	11	3	0.14	0.38	0.07	**0.55**	0.35	0.05	**0.50**	0.10
TRBV10-2	19	9	3	0.17	0.32	0.07	**0.61**	0.28	0.07	**0.55**	0.10
TRBV10-3	93	53	13	0.16	0.20	0.05	**0.75**	0.20	0.04	**0.73**	0.03
TRBV11-2	24	10	3	0.16	**0.61**	0.13	0.26	**0.60**	0.13	0.26	0.01
TRBV11-3	7	3	1	0.09	**0.79**	0.07	0.14	**0.79**	0.07	0.13	0.01
TRBV14	29	10	6	0.28	**0.54**	0.22	0.24	**0.48**	0.19	0.21	0.12
TRBV15	120	55	20	0.42	0.36	0.27	**0.37**	0.33	0.26	**0.36**	0.05
TRBV18	54	26	5	0.20	**0.50**	0.12	0.38	**0.50**	0.12	0.38	0
TRBV19	119	72	44	0.12	0.41	0.06	**0.53**	0.32	0.04	**0.46**	0.18
TRBV20-1	493	291	94	0.39	0.31	0.20	**0.49**	0.27	0.17	**0.46**	0.10
TRBV23-1	14	5	1	0.25	0.39	0.13	**0.48**	0.39	0.13	**0.48**	0
TRBV24-1	55	14	7	0.06	**0.68**	0.06	0.26	0.47	0.05	0.14	0.34
TRBV25-1	32	6	3	0.38	**0.45**	0.26	0.29	0.41	0.22	0.27	0.10
TRBV27	219	49	27	0.22	0.43	0.12	**0.45**	0.35	0.10	**0.39**	0.16
TRBV29-1	5	5	1	0.13	**0.51**	0.08	0.41	**0.49**	0.08	0.40	0.03
5′ end	1	2	3	P_1_	P_0_	P_1_	P_2_	P_0_	P_1_	P_2_	P_3_
TRBD1	3556	4711	2031	0	0.26	0	**0.74**	0.25	0	**0.71**	0.04
TRBD2	1528	1679	724	0.01	0.29	0	**0.71**	0.28	0	**0.68**	0.04
3′ end	1	2	3	P_1_	P_0_	P_1_	P_2_	P_0_	P_1_	P_2_	P_3_
TRBD1	3329	998	202	0	**1.00**	0	0	**1.00**	0	0	0
TRBD2	1680	805	213	0.08	**0.63**	0.03	0.34	**0.63**	0.03	0.33	0.01
	1	2	3	P_1_	P_0_	P_1_	P_2_	P_0_	P_1_	P_2_	P_3_
TRBJ1-1	773	258	96	0.47	**0.42**	0.36	0.22	0.35	0.31	0.20	0.14
TRBJ1-2	150	142	43	0	0.40	0	**0.60**	0.35	0	0.57	0.08
TRBJ1-3	83	107	44	0	**0.97**	0	0.03	**0.97**	0	0.02	0.01
TRBJ1-4	5	1	0	0	**1.00**	0	0	**1.00**	0	0	0
TRBJ1-5	280	248	72	0.40	0.17	0.12	**0.71**	0.17	0.12	**0.71**	0
TRBJ1-6	23	64	26	0	0.14	0	**0.86**	0.12	0	**0.78**	0.10
TRBJ2-1	180	508	200	0	0.18	0	**0.82**	0.18	0	**0.78**	0.04
TRBJ2-2	110	66	25	0.01	**0.89**	0.01	0.10	0.88	0.01	0.09	0.02
TRBJ2-3	442	489	72	0.11	0.21	0.03	**0.76**	0.21	0.03	**0.76**	0
TRBJ2-4	103	82	12	0.45	0.20	0.16	**0.64**	0.20	0.16	**0.64**	0
TRBJ2-5	199	137	38	0.29	0.39	0.15	**0.46**	0.38	0.15	**0.44**	0.03
TRBJ2-6	45	45	15	0	**0.95**	0	0.05	**0.95**	0	0.05	0
TRBJ2-7	301	721	290	0	0.28	0	**0.72**	0.26	0	**0.68**	0.06

* For V gene segments TRBV7-1, TRBV7-4, TRBV7-7, TRBV11-1, TRBV13, TRBV16, TRBV17, TRBV28 and TRBV30 we do not observe any palindrome.

Each entry in the table is the mean of all the donors of all cell types and bold entries in each row represent the maximum conditional probability for a given palindrome. Results are displayed for each identifiable V, D and J gene segments. Columns 2 to 4 show the mean number of one, two and three base observed palindromes in our data set. The probability of no P nucleotide is represented as P_0_.

Several observations can be made from this table. Based on our maximum conditional probability model for classification, we did not predict any single base P nucleotides at any coding end of the V_β_, D_β_, and J_β_ gene segments (**Table 2** **)**. In the case of one base palindromes the average conditional probabilities of one base P nucleotides are 0.22, 0, 0.04, and 0.13 at 3′V_β_, 5′D_β_, 3′D_β_, and 5′J_β_ respectively, which are far below the threshold level of 0.5. Therefore we interpret that one base palindromes arise primarily by the TdT insertion and not by the hairpin resolution. Given an observation of a single base palindrome, we predict with high probability that this base is inserted by the TdT, not a P nucleotide. Furthermore, in two base palindromes the average conditional probabilities of one base P nucleotides at 3′ V_β_, 5′D, 3′D, and 5′J_β_ are 0.15, 0, 0.01, and 0.06 respectively, which are again far below the threshold level of 0.33. The conditional probabilities in three base palindromes behave similar to the two base palindromes case. Most of the two or three base observed palindromes were not classified as one base P nucleotides.

In cases of two and three base observed palindromes, our model predicts either a two base P nucleotide or no P nucleotide. This implies that the third base of the observed palindrome is mostly contributed by the TdT enzyme instead of being part of the hairpin resolution. Most of the two or three base observed palindromes were not classified as one base P nucleotides.

According to the maximum conditional probability model most of the V_β_ gene segments are not predicted to show any P nucleotide (**Table 2** **)**. The classified two base P nucleotides are mainly restricted to few specific V_β_ gene segments (V_β_10 family, V_β_19, V_β_20-1, V_β_23-1, and V_β_27), with two base P nucleotides are dominated by V_β_20-1 and V_β_27 gene segments. We also noticed that there were a few V_β_ gene segments (V_β_7-1, V_β_7-4, V_β_7-7, V_β_11-1, V_β_13, V_β_16, V_β_17, V_β_28 and V_β_30) where no palindromes were observed at all. At 5′D_β_ end, both the D_β_ gene segments were labeled as two-base P nucleotides with high probabilities and the number of sequence with D_β_1 gene segments classified as two-base P nucleotide dominates the D_β_2 by three folds. At 3′D_β_ end, conditional probability was too low for both of the D_β_ gene segments to be classified as P nucleotide. In J_β_ segments, eight of the thirteen J_β_ genes (J_β_1-2, J_β_1-5, J_β_1-6, J_β_2-1, J_β_2-3, J_β_2-4, J_β_2-5 and J_β_2-7) were classified as two base P nucleotides while the remainders of the J_β_ segments were classified as no P nucleotide with high conditional probabilities.

### P Nucleotide Contribution to TCRβ Diversity

Our calculations show that P nucleotides are primarily two bases long. We also observe that if there is a two-base P nucleotides in the TCR sequence, it is mainly seen at one of the four coding ends. Very few sequences have two base P nucleotides at more than one coding end. In each population of T cells, we calculated the mean fraction of sequences which have two base P nucleotides at both ends of N2 or N1 segments (**Table 3**). The mean fraction of two base P nucleotides at both ends of N2 and N1 segments are 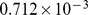 and 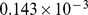 respectively for all cell types. It is not surprise that the second term is smaller than the first one, since the 3′D_β_ gene segments rarely show any P nucleotide. We saw that many of the two base P nucleotides are restricted to the 5′ of D_β_ and J_β_ (**Figure 1B**). We also calculated the mean fraction of sequences which have two base P nucleotides jointly at the 5′ of D_β_ and J_β_. Their mean fractions are 1.4×10^−3^ in all cell types while the CD8^+^ memory has 8.3×10^−4^. These frequencies are hundred times smaller than the total P nucleotide frequencies and therefore we conclude that the formation of two bases P nucleotides at coding ends is statistically a mutually exclusive event and thus the probability that the TCR sequence will have a two-base P nucleotides at more than one coding end will be very small.

**Table 3:** Two base P nucleotides at both ends of N2 and N1 Segment.

T cell population	N2 Segment		N1 Segment		
	Mean	Std	Mean	Std	
CD8^+^ Naïve	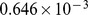	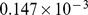	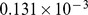	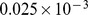	
CD8^+^ Memory	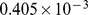	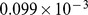	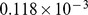	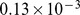	
CD4^+^ Naïve	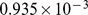	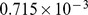	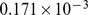	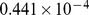	
CD4^+^ Memory	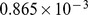	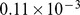	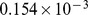	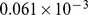	

Number of two base P nucleotides at the both of the N2 segment is calculated in each donor and then normalized by its total number of unique sequences. The entries represent the mean and standard deviation of number of donors in each T cell type. Similarly, it was done for the N1 segment.

We have previously estimated the total number of unique TCRβ CDR3 sequences in the entire T cell repertoire of a healthy adult to be around 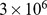 and 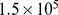 in the naïve and memory compartment respectively [7]. We showed here that total P nucleotide frequency in naïve T cell subsets was approximately 0.02 while the CD8^+^ and CD4^+^ memory compartments has approximately 0.01 and 0.018 respectively (**Figure 1B**), which lead us to estimate using a simple linear extrapolation that approximately 60,000 sequences in the CD8^+^ and CD4^+^ naïve repertoires, 15,000 and 60,000 sequences respectively in CD8^+^ and CD4^+^ memory repertoires would contain P nucleotides of one to three bases long. The frequency of sequences that has two base P nucleotides at one of the four coding ends is found to be around 0.0125 in each cell type while the CD8^+^ memory has 0.008, suggested that around 37,000 sequences would be expected to have two base P nucleotides at any one of the four coding ends in every T cell repertoire except the CD8^+^ memory.

### P Nucleotide Role in Thymic Selection

A subset of T cells has both alleles rearranged. Since we sequence genomic DNA, both the productive and non-productive alleles are observed. For the non-productive alleles, the sequences are either out of frame or have a stop codon. These non-productive alleles account for ∼15% of TCRβ sequences. These alleles are non-functional, and therefore are not subjected to direct selection in the thymus. By comparing properties of these non-productive sequences to the productive sequences in the CD8^+^ naïve repertoire, we are able to deduce which factors contribute to positive and negative selection in the thymus.

**Figure 2:**
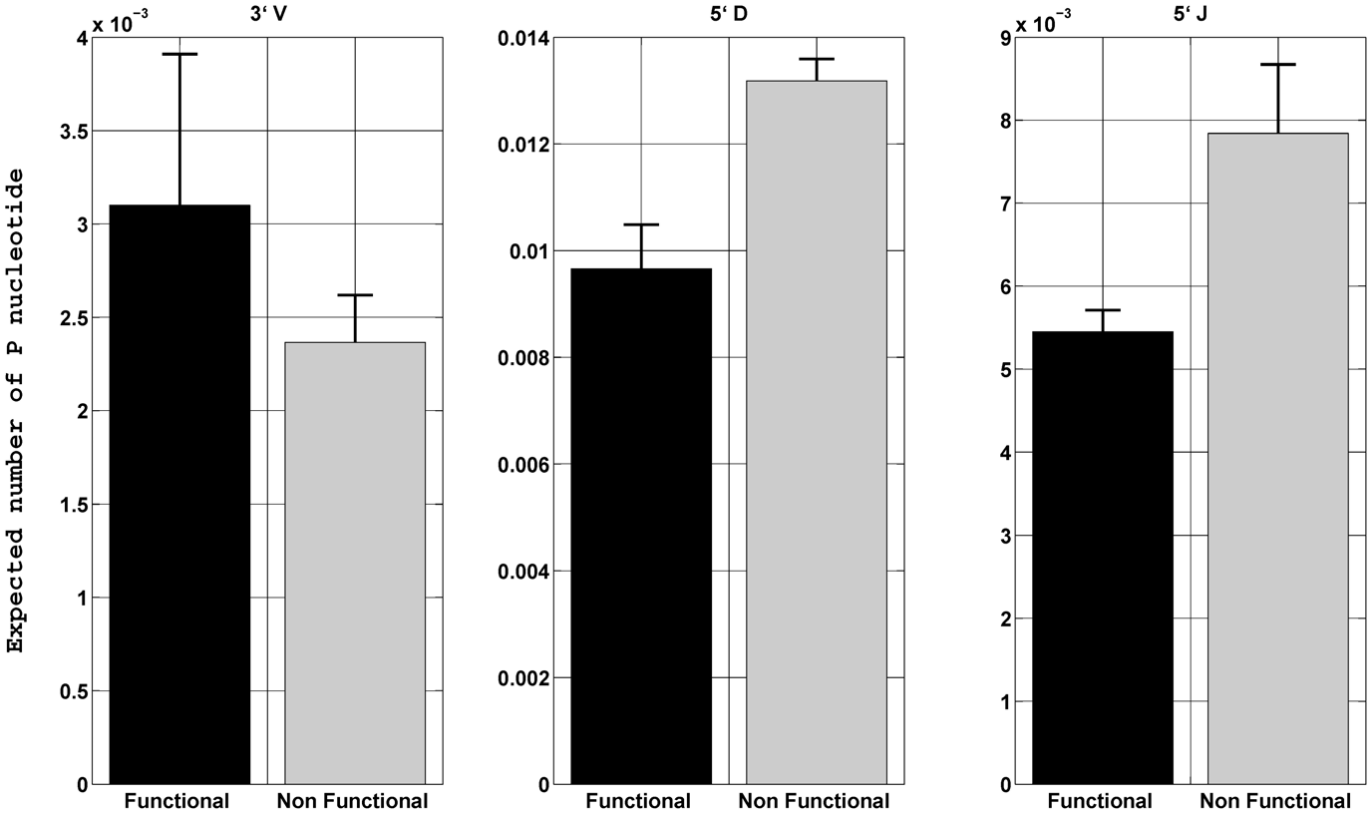
P nucleotide distribution in functional and nonfunctional CD8^+^ naïve T cells at 3′V_β_, 5′D_β_ and 5′J_β_. The height represents the mean of the total (sum of the expected number of one, two and three base) P nucleotides of the seven donors and the error bars indicate one standard deviation.

We calculate and compare the expected number of P nucleotides in productive and non-productive TCRβ sequences at three coding ends, 3′ of V_β_, 5′ of D_β_ and J_β_. We found that non-productive sequences have larger expected number of P nucleotides at 5′ of D_β_ and J_β_ than productive sequences. On the other hand at the 3′ of V_β_, P nucleotides are more predominant in functional TCR sequences than non-functional sequences (**Figure 2**). We showed in our previous work that V_β_−D_β_−J_β_ usage in the functional TCRβ CDR3 sequences was highly non uniform but qualitatively similar to non-functional sequences [6]. In spite of the fact that the V_β_−D_β_−J_β_ utilization is attributed equally in both sets of functional and non-functional TCRβ sequences, we saw statistically significant differences in P nucleotides observed at coding ends in functional and non-functional sequences.

### P Nucleotides Correlate with High Average Copy

We determined that the majority of P nucleotides consist of two nucleotides long in length and they are mainly constrained to the 5′ of D_β_ and J_β_ gene segments (**Figure 1B**). We asked if P nucleotides at these coding ends are associated with large clone size. We take all sequences with no deletion at the specific coding end of the given gene segment and group them as P nucleotide and no P nucleotide depending upon whether a sequence contains P nucleotide greater than one base or not. In order to avoid spurious long P nucleotides at the coding end of the given gene segment, we set the P nucleotide probability of two and three base at the threshold value of 0.05. At each coding end of the gene segment average copy number for the two groups is calculated if the P nucleotide probability at its coding end is found to be above the threshold value. Average copy numbers are normalized by total number of reads in each donor. In each data set we summed the normalized average copy number over D_β_ gene subset and call it the D_β_ sum. Similarly it was done for J_β_ gene subset and J_β_ sum is obtained. We found that all the D_β_ genes contribute to the D_β_ sum while seven to eight J_β_ genes segments contribute to the J_β_ sum.

**Figure 3:**
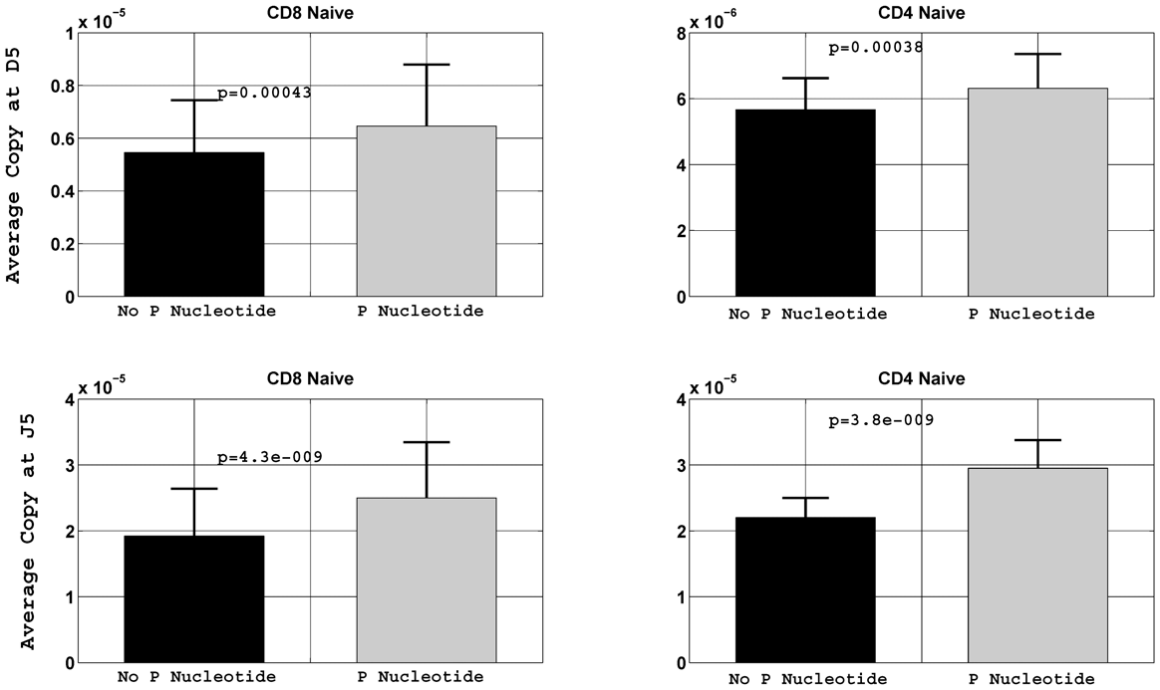
Average copy number with and without P nucleotide. Correlation between average copy number and the P nucleotide at 5′D_β_ and 5′J_β_ gene segment in naïve compartments of the CD8^+^ and CD4^+^ T cells. Heights represent the mean of the sum of the normalized average copy number over D_β_ gene subsets and similarly for J_β_ gene segment subsets. Copy numbers were normalized by respective number of total reads in each donor. The number in each subplot represents the p-value at the significance level of 0.05. Memory compartments of the CD8^+^ and CD4^+^ T cells are shown in the (**Figure S1**). The error bar indicates one standard deviation of number of donors.

We observe that sequences bearing receptors with longer than one base P nucleotides at both the D_β_ and J_β_ coding ends are associated with high average copy number (clone size) as compared to sequences with one or no P nucleotide. Using the Student t-test for paired samples, we found that at the 5′ of D_β_, the two groups are statistically different at the significance level of 0.01 in all T cell compartments except the CD8^+^ memory. On the other hand at the J_β_ end, the two groups are statistically different, using Student t-test for paired samples, in each of the naïve T cell subsets at the significance level of 0.01. Therefore, the distribution is more skewed in the naïve population of T cells in the two groups (**Figure 3**); with longer P nucleotides being biased towards high average copy number. In contrast the distributions in the memory subsets were not as skewed as the naïve (**Figure S1**).

### Characteristic of the Length of Junctional Insertion with P Nucleotide

We showed that a self-complementary nucleotide sequence of two bases was most likely to be a two base P nucleotide. Since the CDR3 length of the TCRβ is constrained, presence or absence of P nucleotides can affect the number of nucleotides added by TdT. We asked if sequences with P nucleotides correlates with junctional insertion. We assess the distribution of the length of the junctional insertion at N2 and N1 segments with and without the two base P nucleotides at either end of the N2 and N1 segments.

To compare the number of junctional insertions at N2 and N1 segments of the TCRβ CDR3 with P nucleotides (consisting of more than one base) and no P nucleotide sequence, we partition the data set from each donor into two groups. We call a sequence a P nucleotide sequence if we observe a palidrome greater than one base at either end of the N2 segment otherwise as no P nucleotide sequence. As mentioned above we observe very few P nucleotides at both ends of N2. For each P nucleotide and no P nucleotide sequence we calculate the length of nucleotides inserted by the TdT enzyme. Note that to calculate the length of nucleotides inserted by TdT in P nucleotide defined sequences, we need to factor out the P nucleotide length from the total insertion, since these P nucleotide bases are not inserted by the TdT enzyme. The calculation was repeated for the N1 segment.

**Figure 4:**
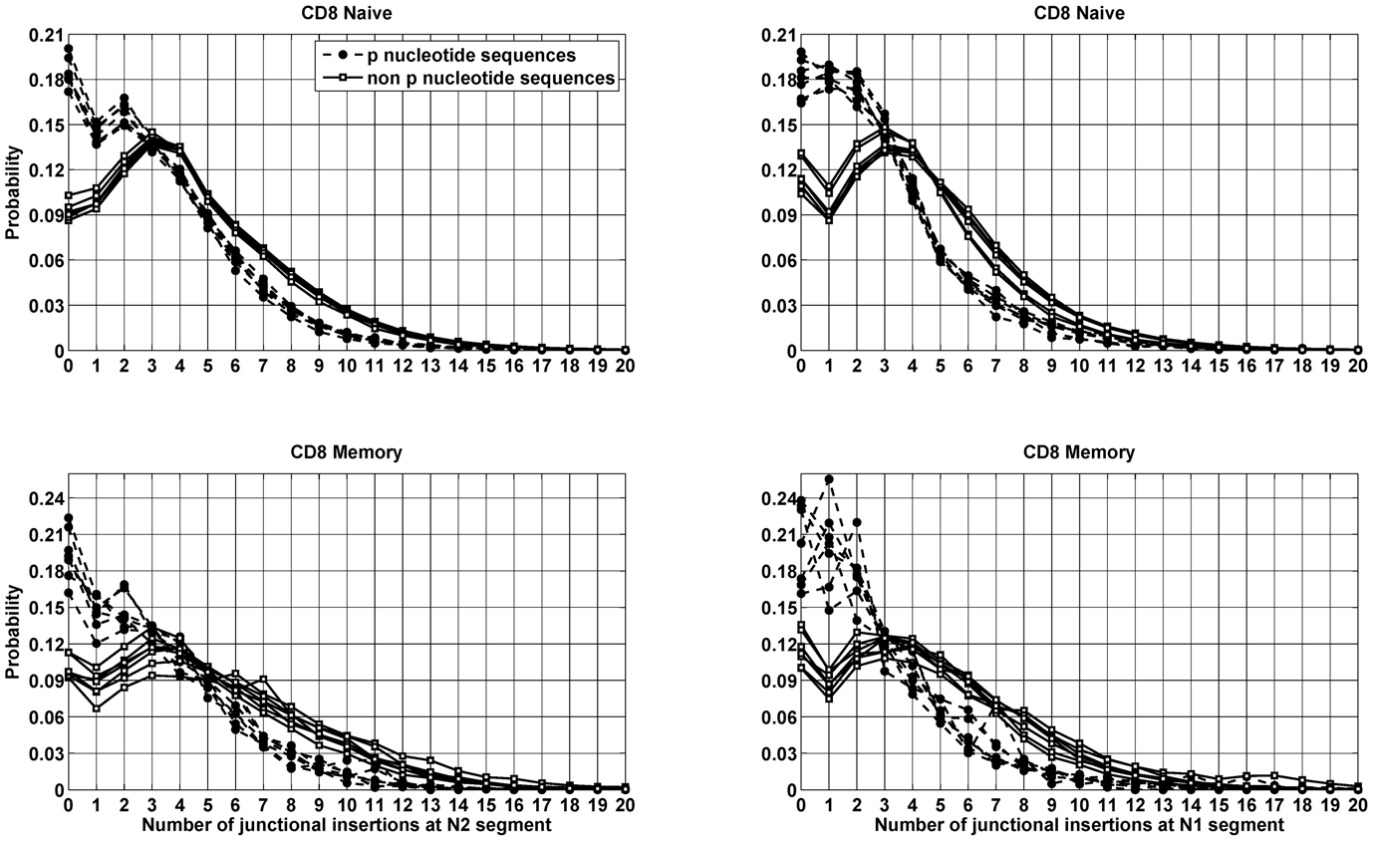
Insertion distribution of P nucleotide sequence. Insertion distribution of P nucleotide and no P nucleotide CDR3 sequences observed in the naïve and memory CD8^+^ compartments of every possible pair of individuals as a function of the number of nucleotide insertions at the N2 and N1 segments. CD4^+^ T cells also show a similar distribution, (**Figure S2**).

The results show that CDR3 sequences with P nucleotides consisting of more than one nucleotide are likely to have fewer non-template insertions as compared to sequences with no P nucleotide in the naïve and memory compartment of the CD8^+^ and CD4^+^ T cells. Results are shown for the CD8^+^ T cells, (**Figure 4**) and the CD4^+^ T cells show a similar trend (**Figure S2**). It is interesting to note that the two distributions intersect each others when the number of insertions becomes equal to three and this is consistent in each T cell compartment. Thus the probability of up to three insertions is higher in the P nucleotide sequences as compared to the no P nucleotide sequences. On average P nucleotide sequences have three nucleotide insertions in both the naïve and memory compartments while no P nucleotide sequences were found to have four and five nucleotide insertions in naïve and memory compartments respectively. This makes the P nucleotide sequences one to two bases closer to the germ line as compared to the no P nucleotide sequences. And thus the addition of P nucleotides consisting of two bases can reduce the diversity of TCRβ repertoire up to 64 fold.

### P Nucleotide Induces Reading Frame Bias

D_β_ gene segments of the TCRβ can be transcribed in all three reading frames. In order to see if the addition of a P nucleotide is biased toward or against a particular reading frame in the D_β_ gene segments reading frames 1, 2 and 3 were defined if the 1^st^ undeleted nucleotide of the 5′ end of the D_β_ gene segment was at the 1^st^, 2^nd^ and 3^rd^ base of the codon respectively.

Comparing the effects of P nucleotides on the reading frame, we found that the addition of P nucleotides at the 5′ end of the D_β_ gene segments increased the frequency of the reading frame 1 in all populations of T cells. When a P nucleotide was inserted, 50–60% of T cells used reading frame 1 to transcribe their TCRβ chains. Sequences with the addition of two base P nucleotides in this reading frame, the first position of the codon is occupied by any nucleotide and the two bases of the P nucleotide contribute to the non-synonymous and the synonymous position of the codon. Thus the consequences of two base P nucleotide in this reading frame leads to four canonical amino acid residues, alanine, proline, serine, or threonine in the CDR3β sequences depending upon if the first position of the codon is occupied by G, C, T or A respectively. The respective frequency of the amino acid residues in all T cell subtype is given in **Table 4**. Proline and threonine were coded more than uniformly while alanine was less than uniform in this reading frame.

**Table 4:** Amino acid usages in reading frame 1.

T cellpopulation	Alanine	Proline	Serine	Threonine
CD8^+^ Naïve	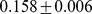	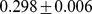	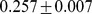	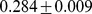
CD8^+^ Memory	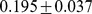	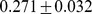	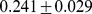	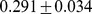
CD4^+^ Naïve and Memory	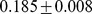	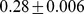	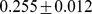	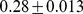

Frequency distribution of amino acid distribution with two base P nucleotides in the reading frame 1. Proline and threonine are coded more than uniform while alanine is coded less than uniform. Each entry in the table is the mean frequency with the standard deviation.

The third reading frame with P nucleotides was favored slightly less as compared to no P nucleotide. But note that in this reading frame, sequences with no P nucleotide could add many possible amino acids at this position, while the same reading frame with P nucleotide would only add a proline reside since the two bases of the P nucleotide only affect the non-synonymous positions of the codon. This means that 30–40% of sequences with P nucleotides in this reading will only have proline followed by glycine and thus suggested that these residues might play some important biological role in TCR recognition. In the P nucleotide sequences the combined affect of reading frames 1 and 3 at the amino acid level introduce a strong bias for proline which is the only cyclic amino acid and allows less degrees of freedom.

**Figure 5:**
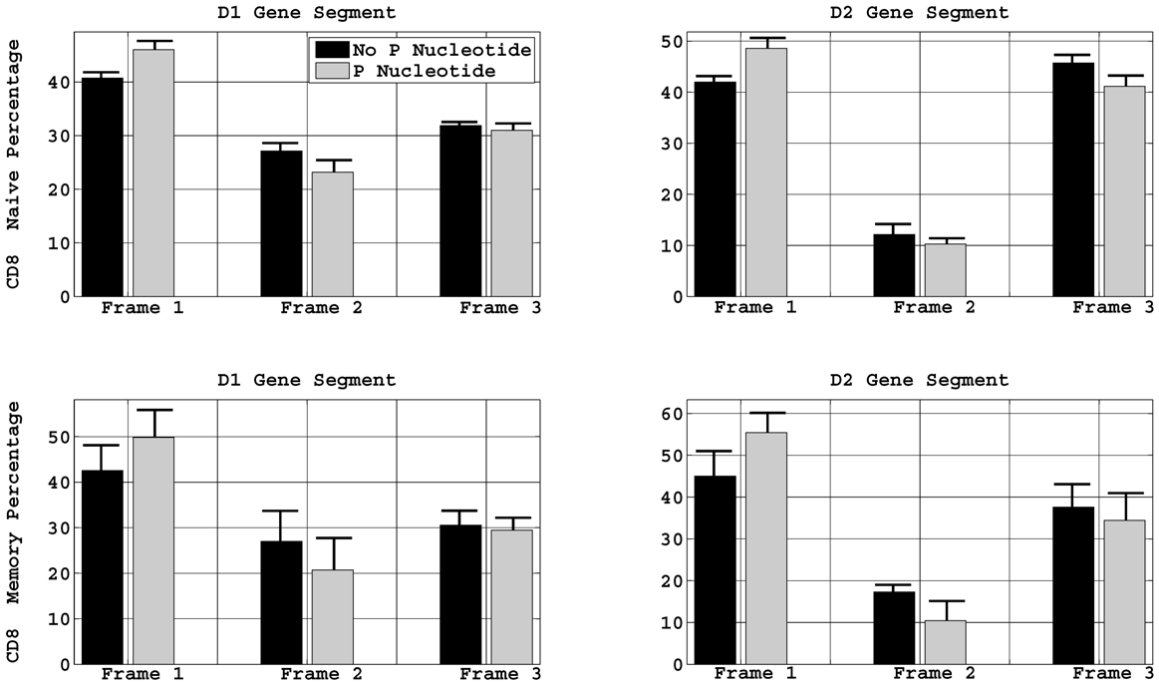
Reading frame biased induced by P nucleotide. Heights represent the mean percentage of sequence with a reading frame in P nucleotide and none P nucleotide. Results are shown for D_β_1 and D_β_2 gene segments in the CD8^+^ naïve and memory T cells. Naïve and memory CD4^+^ T cells also show a similar distribution, (**Figure S3**). Error bars indicate the standard deviation.

We found that irrespective of the P nucleotides, the frequency of the D_β_1 segment in the reading frame 2 was higher than the D_β_2 segment, 25% and 10% in D_β_1 and D_β_2 segments respectively in all T cell populations. Thus, when T cells rearrange their DNA, they are less likely to use D_β_2 segment to encode TCRβ chains in the 2^nd^ reading frame and the addition of P nucleotides further decrease this usage (**Figure 5**). Furthermore D_β_2 gene segments also contain a stop codon “TAG” and we observed that whenever the D_β_2 segment was used in the 2^nd^ reading frame, eleven nucleotides were invariably deleted from its 3′ to avoid this stop codon. This also explained the lower number of P nucleotide additions at the 5′ end of D_β_2 as compared to D_β_1. Thus, the P nucleotide addition in this reading frame only leads to incorporation of arginine followed by aspartic acid in the CDR3 TCRβ.

## Discussion

High-throughput DNA sequencing has enabled us to characterizes P nucleotide distributions in millions of distinct TCRβ CDR3 sequences found in multiple individuals. We develop a novel algorithm to calculate P nucleotide at full-length coding ends in TCR. This algorithm allows us to calculate the probability of an observed palindromic nucleotide being truly P nucleotide as opposed to be inserted by the TdT as a palindromic N-nucleotide. We find that average length of P nucleotide is 2 and the range is 1 to 3 (**Figure 1B**). These findings are consistent with biochemistry of the Artemis:DNA-PKcs in V_H_ segments of human immunoglobulin heavy chain locus, which has a distribution of hairpin opening position that varies from 1 to 3 nucleotide 3′ of the hairpin tip, with 2 nucleotide being the dominant hairpin opening site [9], [10]. Our results are also in agreement with the works of two different authors [3], [4], where they investigated the P nucleotide using extrachromosomal plasmid assay and also performed an in-depth analysis of two loci, the TCRβ locus and the IgH locus. They found that 95% of P nucleotides (palindromes in our terminology) were one to two residues in length and 5% were 3 bp long. These agreements allow us to conclude that the length distribution of P nucleotides could also be generalized to other loci.

The P nucleotide distribution profile is biased in two ways. First, it favors the P nucleotide insertions at certain coding ends, 5′ of D_β_ and J_β_ (**Figure 1B**). Second, at the coding end it is further biased with respect to the gene sequence used, some gene sequences display high proportion of P nucleotides while others show few or none (**Table 2** **)**. The 3′V_β_ coding end P nucleotides are dominated by V_β_20-1 and V_β_27 gene segments. The coding end of these two gene segments are A/T rich and therefore TdT bias for G/C couldn’t account for these differences. However, the observed frequencies of V_β_–J_β_ utilization suggest that these segments are predominantly used in all subsets of T cell [6], and thus either positive or negative selection could biased the samples. Parallel with this, Meier and Lewis’s work also reported that the palindrome frequency data associated with 3′V_β_ coding end were dominated by a single V_β_ gene segment. The 5′J_β_ coding end P nucleotides are mainly observed in the members of the J_β_2 family (**Figure 1B**), and in the case of 2 and 3 base palindrome data they are classified as 2 base P nucleotides with high conditional probabilities (**Table 2** **)**.

The total palindrome percentage at the coding end ranges from 2 to 6% (**Figure 1A**), while the total P nucleotide percentage, sum over all the coding ends, ranges from 1–2% in the CD8^+^ and CD4^+^ naïve T cells, with less or so in the memory T cells (**Figure 1B**). We compared our results with Meier and Lewis’s work that used the substrate assay to analyze palindrome formation at coding joints [3]. They reported that percentage of palindromes at the coding joint ranges from 3% to 16%, see their Table 2, which is higher than our result. This difference is expected and could be accounted by considering the fact that their substrates were analyzed without selective bias, positive and negative selection, of an intact adaptive immune system and thus their estimated palindrome frequencies could be overestimated. It is estimated that 70–80% of the rearranged T cells die during the selection processes of the VDJ recombination [11]. If we consider this fact, Meier and Lewis’s estimates could be scaled down and become agreeable to our result. They also comprehensively revaluated a few hundred sequences from previously published studies; see their Table 3, [3]. They showed that the palindrome frequencies in TCRβ locus at 3′V_β_, 5′D_β_, 3′D_β_, and 5′J_β_ are 0.1, 0.15, 0.04, and 0.11 respectively, which are approximately 2–3 folds higher than our result (**Figure 1B**). Although TCRβ genes with P nucleotides constitute a small fraction of the total repertoire, they are observed consistently in our cohort and have evidence of strong selection.

According to the data shown (**Figure 1A**), 1–2% of the total unique sequences have single base palindromes at each coding end. We computed the conditional probability of P nucleotide using an algorithm outlined in Materials and Methods. As detailed in (**Table 2** **)** classification of P nucleotides based on maximum conditional probability, in the case of one base palindrome, did not predict any single base P nucleotides at any coding end of the V_β_, D_β_ and J_β_ gene segments. This result supports the view that the TdT is mainly responsible for single base palindromes. An alternative possibility is that one base palindromes were actually a 5′ and 3′ palindromic overhangs of ≥2 nucleotides long in length, and they could go through exonuclease trimming by Artemis protein to yield palindromes of single base. It has been shown that the purified Artemis protein possesses a single-strand-specific 5′ to 3′ exonuclease activity [10]. To more fully characterize the single base palindromes, we computed the nucleotide frequency, composition, of the single base palindromes as well as the average mono nucleotide frequency of the TdT insertions (**Table 5**). Though TdT has a coding end bias (**Table S3**), on average it has a strong well-known bias for inserting G/C nucleotides [4]. The result showed that the nucleotide frequency of single base palindromes is overrepresented at the coding ends terminating in G/C as compared to A/T, and this could be explained by the TdT’s bias for inserting palindromic G/C nucleotides.

**Table 5:** Overrepresentation of one-base palindromes at coding ends terminating in G/C.

T cell population		Frequency			
		A	C	G	T
CD8^+^ naïve	TdT	0.205	0.286	0.284	0.225
	1-base palindrome	0.063	0.415	0.357	0.165
CD8^+^ Memory	TdT	0.231	0.236	0.319	0.214
	1-base palindrome	0.061	0.401	0.395	0.143
CD4^+^ naïve	TdT	0.2	0.292	0.298	0.210
	1-base palindrome	0.067	0.432	0.338	0.163
CD4^+^ Memory	TdT	0.204	0.282	0.305	0.209
	1-base palindrome	0.062	0.434	0.336	0.168

Nucleotide frequency of the single base palindromes is compared with the mono nucleotide distribution of the TdT insertions. One-base palindromes is overrepresented at the coding ends terminating in G/C as compared to A/T, and this difference could be explained by the TdT’s bias for inserting palindromic G/C nucleotides. Each entry in the table is the mean frequency.

In the CD8^+^ naïve T cells three donors are related, donors 1 and 3 are siblings and shared the same HLA, they have different age, and are the daughters of donor 2, while the other four donors are completely unrelated in HLA and belong to different age groups. At each coding end we computed the total fraction of palindromes of 1–7 base long in each donor. The fraction of palindromic inserts varied, but no systematic difference emerged (**Table S4**), though this data set is small to make any general conclusion. We observed that there is no variation in palindromic insertion at the 5′J_β_ with regard to the HLA. We saw some HLA-related differences at the 3′V_β_, the two related siblings shows similar fraction of palindromes, donors of African origin show higher fraction of palindrome insertions. One might expect a variation in palindromes due to HLA as the V_β_ gene segments interact with the HLA as well as with the peptide for antigen recognition.

The significant differences between P nucleotide usage in productive and non-productive naïve TCRβ sequences suggests strong thymic selection (**Figure 2**). The error bars in Figure 2 are standard deviation. The standard errors are the STD/sqrt(7), which implies that P nucleotide frequency differences in the productive and non-productive sequences are statistically significant (P<.01). P nucleotides fix particular amino acids at specific positions in the CDR3 sequence, so thymic selection is expected **(** **Figure 5** **)**. However, the 30–50% observed is very large. Most other sequence properties, such as CDR3 length and GC content, are indistinguishable between productive and non-productive TCRs, and show little sign of influencing thymic selection. These differences are observed for P nucleotides for V_β_, J_β_, and 5′D_β_.

On the other hand, there are very few P nucleotides at the 3′D_β_ end in either naïve or memory compartments of CD8^+^ and CD4^+^ T cells (**Figure 1B**). As this observation holds for both productive and non-productive, thymic selection is not a viable explanation. Another possibility is the strength of the RSS signal [11]. The 3′D_β_ 23 RSS mediates joining to J_β_ 12 RSS more efficiently than V_β_ 23 RSS does to 5′D_β_ 12 RSS and thus we hypothesized that efficient joining could give rise to fewer P nucleotides at the coding end.

**Figure 6:**
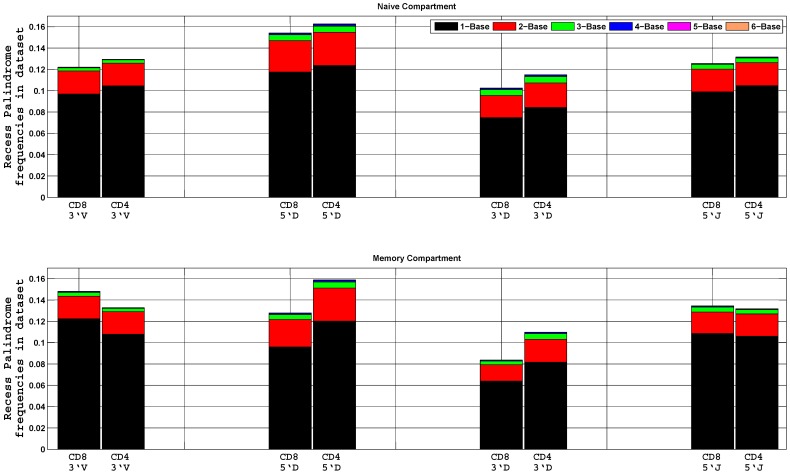
Palindrome length frequencies at recessed coding ends. Histogram of the average palindrome length frequencies at the four recessed coding ends in the data set of naïve and memory compartments of the CD8^+^ and CD4^+^ T cells. Each entry is normalized by its total number of unique sequences. The mean height is shown in the figure. Different colors represent one to six bases palindromes. In contrast to the palindromes at non-recessed coding ends, events of one base palindrome are more common at nucleolytically processed coding termini.

This manuscript is based on the analysis of palindromes at the end of non-recessed coding ends. For the completion, we also briefly report the presence of another kind of palindromes at nucleolytically processed coding termini, referred by Gauss and Lieber as Pr nucleotides (referred to here as recessed palindromes) [4]. Although, the biological process that gives rise to these Pr nucleotides is not clear, the authors have hypothesized that their generation could be attributed to either fortuitous addition by the TdT enzyme or by a hairpin intermediate. Histogram of the recessed palindrome length frequencies, based on 1 to 10 nucleotide deletions at coding termini, is shown in **(** **Figure 6**). In contrast to the conventional palindromes which are on average 2 nt in length (**Figure 1A**), the frequency of a single nucleotide recessed palindromes is over-represented. We find that 98–99% of the recessed palindromes are 1 to 3 bases long, and the average length is 1.2, which are consistent with the results of Gauss and Lieber [4].

With the algorithm presented to assign a probability that an observed palindrome is truly a P nucleotide, we can begin to study the functional role of P nucleotides [12], [13]. Functional immunological assays such as tetramer sorting enables the identification of the subset of T cells which bind a particular HLA:peptide antigen complex. With an assay, we are able to readily sequence the set of these TCR sequences. Combining such functional assays with the characterization of P nucleotides presented here, we can elucidate the function role TCRs with P nucleotides in different disease contexts.

## Materials and Methods

### Notations and Terminology

For our purposes, and in keeping with previous works, we introduced some notations and terminologies. We denoted the nucleotide insertion at D_β_−J_β_ and V_β_−D_β_ junctions as N1 and N2 regions respectively. We denoted the observed and true complementary sequence as palindrome and P nucleotide respectively. The probability of i-base P nucleotide is represented as p_i_. “Coding” end referred to the sequence directly adjacent to the recombination signal sequences (RSS). TCRβ CDR3 region is defined as a sequence that begins at the nucleotide base that encodes the 2^nd^ conserved cysteine at the 3′ of the V_β_ gene and ends at the nucleotide base that encodes the conserved phenylalanine at the 5′ of the J_β_ gene.

### Isolation and Purification of Naïve and Memory CD8^+^ and CD4^+^T Cells

Isolation and purification of naïve and memory compartments of the CD8^+^ and CD4^+^ T cell samples were carried out as described in [6], [7].

### Sequencing of CDR3 Regions from Rearranged TCRβ Genes

Polymerase chain reaction (PCR) amplification and sequencing of rearranged TCRβ CDR3 regions, in reverse complement from 3′J_β_, were done as described in [6], and see also Figure 1 in the reference [7].

### Preprocessing of Genome Analyzer (GA) Sequence Data

Raw GA sequence data, 60-nucleotide long, were preprocessed to remove PCR and sequencing errors. Clustering of data into unique TCRβ CDR3 sequences were done using Hamming distance, as described in [6], [7]. The sequence data is again reverse complemented to bring them back into 3′V_β_ to 5′J_β_ orientation.

### Identification of TCRβ CDR3 Sequences and VDJ Decomposition

Identification of the TCRβ CDR3 region and the nomenclature were done according to the definition established by the International ImMunoGeneTics collaboration [8]. Accurate decomposition of TCRβ CDR3 nucleotide sequence into constitutive gene segments represents a major challenge for P nucleotide analysis. Each nucleotide of the solexa sequence is assigned to the most likely gene segment. An updated IMGT algorithm was used to delimit the 5′ end of J_β_, the 3′ end of the V_β_, and both the ends of the D_β_ by aligning the solexa sequence with the reference genes. Since somatic hypermutation do not occur in T cell, any difference between the reference gene and solexa sequence is not allowed in the CDR3 region and therefore the first nucleotide difference determine the end of the gene segment.

Each of the 13 different J_β_ gene segment has a unique nucleotide motif between the position 12 and 18 from the heptamer signal sequence. The last several bases of solexa sequence are searched for J_β_ gene specific J motif, this in turn uniquely determined which J_β_ segment was used. If none of the J motif was found in the solexa sequence, the sequence was not considered for P nucleotide analysis. If the J motif is found, solexa sequence is compared base by base, 3′ to 5′ orientation, to the reference J_β_ gene segment. Truncated or nontruncated 5′J_β_ coding end is respectively determined by the next nucleotide difference or by the end of reference J_β_ gene segment.

Each of the 54 V_β_ gene segment has a 7-base nucleotide motif which encodes the first four amino acid residues “CASS” of the CDR3. Solexa sequence is searched for V_β_ gene specific motif. If V_β_ gene specific motif was not found for any of the V_β_ gene, the solexa sequence was dropped from the analysis. If the motif is found, solexa sequence is divided into two subsequences: 1^st^ subsequence is the sequence downstream of the motif, containing the motif, and the 2^nd^ subsequence is upstream of the motif. Since the V_β_ part of the CDR3 region is too short, V_β_ assignment is done in two steps, unlike the original IMGT algorithm. In the first step truncated or nontruncated 3′V_β_ coding end, thus the number of undeleted V_β_ nucleotides in the solexa sequence, is determined by comparing the 1^st^ subsequence, nucleotide by nucleotide in 5′ to 3′ orientation, to the CDR3 part of the reference V_β_ gene segment. In the second step upstream similarity score was calculated for each reference V_β_ gene, without gap, using a sliding window between the 2^nd^ subsequence and upstream sequence of the CDR3 part of the reference V_β_ gene. The number of undeleted nucleotides calculated in step 1 was added to the upstream similarity score; adding this creates more weight to the CDR3 part of the nucleotides of the V_β_ gene segment. If the V_β_ segment in the solexa sequence is more likely to be close to the full coding end more likely it is to be uniquely defined. The V_β_ gene corresponding to the maximum score is assigned to the solexa sequence. If the maximum score is not unique, V_β_ gene cannot be assigned uniquely to the solexa sequence, the solexa sequence is dropped from the analysis.

After parsing V and J, the nucleotide sequence between 3′V_β_ and 5′J_β_ termini is compared with the reference D_β_ gene segments. For each D_β_ gene similarity score was calculated, without gap, using a sliding window between the nucleotide sequence and the reference D_β_ gene. The D_β_ gene corresponding to the maximum score is assigned to the sequence and is used to delimit both the ends of the D_β_ segment using the procedure described above for V and J. If the D_β_ gene segment in the solexa sequence is not uniquely assigned or the number of undeleted nucleotides in the D_β_ part of the solexa sequence is less than 5, the solexa sequence is dropped and not considered for P nucleotide analysis.

At each indentified and delimited full-length coding end, 3′V_β_, 5′D_β_, 3′D_β_, 5′J_β_, the algorithm search for short palindromic sequences. The algorithm starts the search from the last (first) nucleotide of the gene segment at the 3′(5′) coding end and compared it with the first (last) nucleotide downstream to the 3′(5′) coding end. If the comparison is found to be complementary, the iteration moves to the second last (second) nucleotide of the gene segment and compared it with the second (second last) nucleotide downstream to 3′(5′), and so on. The iteration stops when the next nucleotide comparison is not found to be complementary. Number of iterations determines the length of the palindrome. After palindromic nucleotides are identified they are excluded from contributing to the upstream or downstream gene sequences that remained to be queried. N1 and N2 regions are the part of the sequence that left after the identification and delimitation of the V_β_, D_β_, J_β_ and palindromic nucleotides.

### Estimation of P Nucleotide Probability

At the coding ends, P nucleotides are followed by random insertions by the TdT (N nucleotides). There is no well-defined boundary between the P and N nucleotides to decompose them into disjoint sequences. Observed palindromes can be explained purely by true P nucleotides or by N nucleotides, or by a joint combination of both. We establish a linear system to model the P nucleotide probabilities, in which each equation quantifies the frequency of observed palindromic sequence (less than equal to 3 nts) as a combination of P nucleotide and N nucleotide distributions.

Let 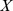 be a random variable over the P nucleotide length, whose probability distribution is denoted by 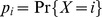, where 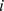 takes positive integer values and 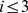. Let 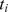 be the probability that the 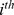 base with respect to the coding end is inserted by the TdT. The observed frequency 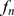 that the first 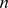 nucleotides are self-complementary or palindromic is equal to the sum of the probabilities that these nucleotides are independently added by the TdT or the combination of P nucleotide and TdT or has at least 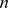
- base P nucleotide. This can be expressed mathematically as
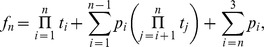
where 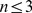. In the above equation, the first term represents the probability that all 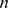 nucleotides are independently added by the TdT, the middle term is the probability that the first 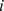 bases are P nucleotide and the remaining 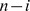 bases are N insertions, and the last term represents the probability that there is at least 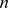-base P nucleotide. By denoting 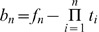, the above equations become a system of three equations with three unknown’s **p** = 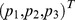. In a matrix form, it is written as



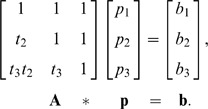



This system suggests that in order for a sequence to have a P nucleotide, we must factor out the TdT likelihood from the observed palindrome frequencies. The column vector **b** reflects that excess probability, after subtracting the TdT’s bias for inserting certain nucleotides, which cannot be explained by TdT, and thus it contributes to the true formation of P nucleotides. Given the vector **b** from the data and TdT probabilities, **p** is uniquely solvable by inverting the matrix **A**. Since the TdT probabilities are greater than zero, the matrix **A** is positive definite and hence invertible.

The solution of the above system must satisfy the following conditions. 1) Since the left hand side of each equation is the sum of probability terms, **b** must satisfy the constraint **b** ≥ **0**. 2). If 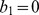, it implies that **p = 0**. This is equivalent to saying that if the first base is added by the TdT to the coding end, it is not possible for the coding end to have a P nucleotide. 3) Since 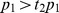, comparing the first and second equations gives 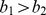. In order to have a one base P nucleotide this condition must hold. 4) Again since 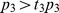, comparing the second and the third equations gives 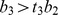. This inequality implies that in order to have a three base P nucleotide, the information content of P nucleotide in three base pairs must be greater than the combined information of two base pairs and the information that the 3^rd^ base pair is inserted by the TdT. 5) As a special case, if no nucleotide is added by the TdT, P nucleotide probabilities trivially become 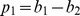, 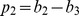, 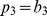, i.e. all the palindromic sequences are P nucleotides.

In order to solve for **p,** the above system requires the estimation of the entries of the matrix which depend only on the TdT probabilities and the vector **b** which are dependent upon the TdT probabilities as well as on empirical frequencies of observed palindromes. We calculated the empirical frequencies of the palindromes based on the data. Estimation of the TdT probabilities is described in the section Method Overview. Mean estimated TdT probabilities for the CD8^+^ and CD4^+^ T cells are given in **Table S3**. We substituted the coding end specific TdT probabilities and the empirical frequencies of palindromes into the above system and solve for P nucleotide probabilities of different length.

### Algorithm for Assigning P Nucleotide Class

Many palindromic sequences appear at V_β_−D_β_ or D_β_−J_β_ junctions that are derived in whole or in part by nucleotide insertions from the TdT. Classifying all of the n-base palindromes into n-base P nucleotides overestimate the P nucleotide’s distribution profile and underestimate the TdT distribution in the data set. Here we take an approach of classifying a given palindrome into a P nucleotide of certain length using a maximum conditional probability model. For a 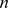 base palindrome at a given coding end we calculated the conditional probability of P nucleotide of 1 to 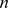 length, and the conditional probability of no P nucleotide and then chose the P nucleotide class that has the largest conditional probability.

Let 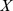 and 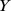 be random variables over the length of a P nucleotide and an observed palindrome respectively. Using a Baye’s rule, given a 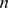 base palindrome, the conditional probability of a 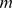-base P nucleotide is written as.
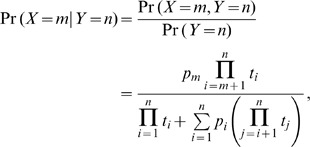
where 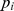 is the 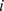-base P nucleotide probability, 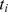 is the probability that the 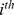 base with respect to the coding end is a TdT insertion and 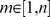. The conditional probability of no P nucleotide is calculated using the expression 
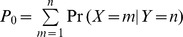
. P nucleotide probabilities 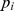 at each coding end are estimated using the linear system outlined in Materials and Methods, and the TdT probabilities 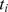 are estimated as described in Method Overview and theirs estimates are given in **Table S3**. In the above equation the denominator captures the number of possible ways a 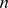 base palindrome arises while the numerator expresses the probability of the event that a 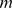 base P nucleotide is followed by 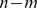 bases of TdT insertions. For example, if we observed a two base palindrome at any given coding end, the conditional probability of 0, 1 and 2 base P nucleotide become 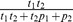, 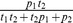 and 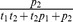 respectively. Thus, we classified the two-base palindrome as a 0-base, no P nucleotide, or 1-base, or 2-base P nucleotide corresponding to the term which maximizes the conditional probability.

### Statistical and Computational Analysis

In each donor and at the each coding end, the expected number of P nucleotide was calculated using the expression 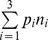, where 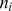 represents the number of observed i-base palindrome and 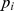 is the probability of i-base P nucleotide, estimated using the linear system described in Materials and Methods. The 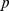
values were determined by the Student 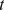-test for paired samples. All bar heights are shown as group means and the vertical error bars represent one standard deviation. All computations are performed in Java and Matlab.

## Supporting Information

Figure S1 Average copy number with and without P nucleotide. Correlation between average copy number and P nucleotide at 5′ D_β_ and 5′ J_β_ gene segment in memory compartments of CD8^+^ and CD4^+^ T cells. Heights represent the mean of the sum of the normalized average copy number over D_β_ gene subsets and similarly for J_β_ gene segment subsets. Copy numbers were normalized by respective number of total reads in each donor. The error bar indicates one standard deviation.
(TIF)

Figure S2 Number of insertions distribution in P nucleotide sequence. Insertion distribution of P nucleotide and no P nucleotide CDR3 sequences observed in the naïve and memory CD4^+^ compartments of every possible pair of individuals as a function of the number of nucleotide insertions at the N2 and N1 segments.
(TIF)

Figure S3 Reading frame biased induced by P nucleotide. Heights represent the mean percentage of sequence with a reading frame in P nucleotide and none P nucleotide. Results are shown for D_β_1 and D_β_2 gene segments in CD4^+^ naïve and memory T cells. Error bars indicate the standard deviation.
(TIF)

Table S1 Summary of CD8^+^ naïve and memory data set. Number of in-frame, TCRβ CDR3 nucleotide sequence, total reads and unique number of sequence obtained from the CD8**^+^**CD45RO**^−^**CD45RA**^hi^**CD62L**^+^** (naïve) and CD8**^+^**CD45RO**^+^**CD45RA**^low^** (memory) T-cell samples from each donor are given in the second and third columns respectively. The corresponding number of sequence with untrimmed coding ends at 3′V_β_, 5′D_β_, 3′D_β_, and 5′J_β_ are shown in the last four columns. Each row shows the naïve (above) and memory (below) data.
(DOC)

Table S2 Summary of CD4^+^ naïve and memory data set. Number of in-frame, TCRβ CDR3 nucleotide sequence, total reads and unique number of sequence obtained from the CD4**^+^**CD45RO**^−^**CD45RA**^hi^**CD62L**^+^** (naïve) and CD4**^+^**CD45RO**^+^**CD45RA**^low^** (memory) T-cell samples from each of the six donors are given in second and third columns respectively. The corresponding number of sequence with untrimmed coding ends at 3′V_β_, 5′D_β_, 3′D_β_, and 5′J_β_ are shown in the last four columns. Each row shows the naïve (above) and memory (below) data.
(DOC)

Table S3 TdT probabilities estimate for all cell types at each of the four coding ends. Probability estimate of N nucleotide insertion is shown based on independent assumption. In order to avoid any potential bias middle regions of N2 and N1 segments are considered for the calculation of average nucleotide frequency, described in details in Methods Overview.
(DOC)

Table S4 Demographic characteristic of palindromic sequences. In each donor the fraction of palindromic inserts of up to six bases long is shown against the coding ends in CD8^+^ naïve T cells. The 5′J_β_ does not show any variation while the 3′V_β_ shows some fluctuations with regard to the HLA. The two related siblings, donor 1 and donor 3, show the similar pattern of palindromes at 3′V_β_, 5′D_β_ and 5′J_β_ coding ends.
(DOC)
