# Supplementary figures and images for: Palindromic Nucleotide Analysis in Human T Cell Receptor Rearrangements
Source: PLoS One. 2012 Dec 21;7(12):e52250. doi: 10.1371/journal.pone.0052250 (PMC3528771; doi:10.1371/journal.pone.0052250)

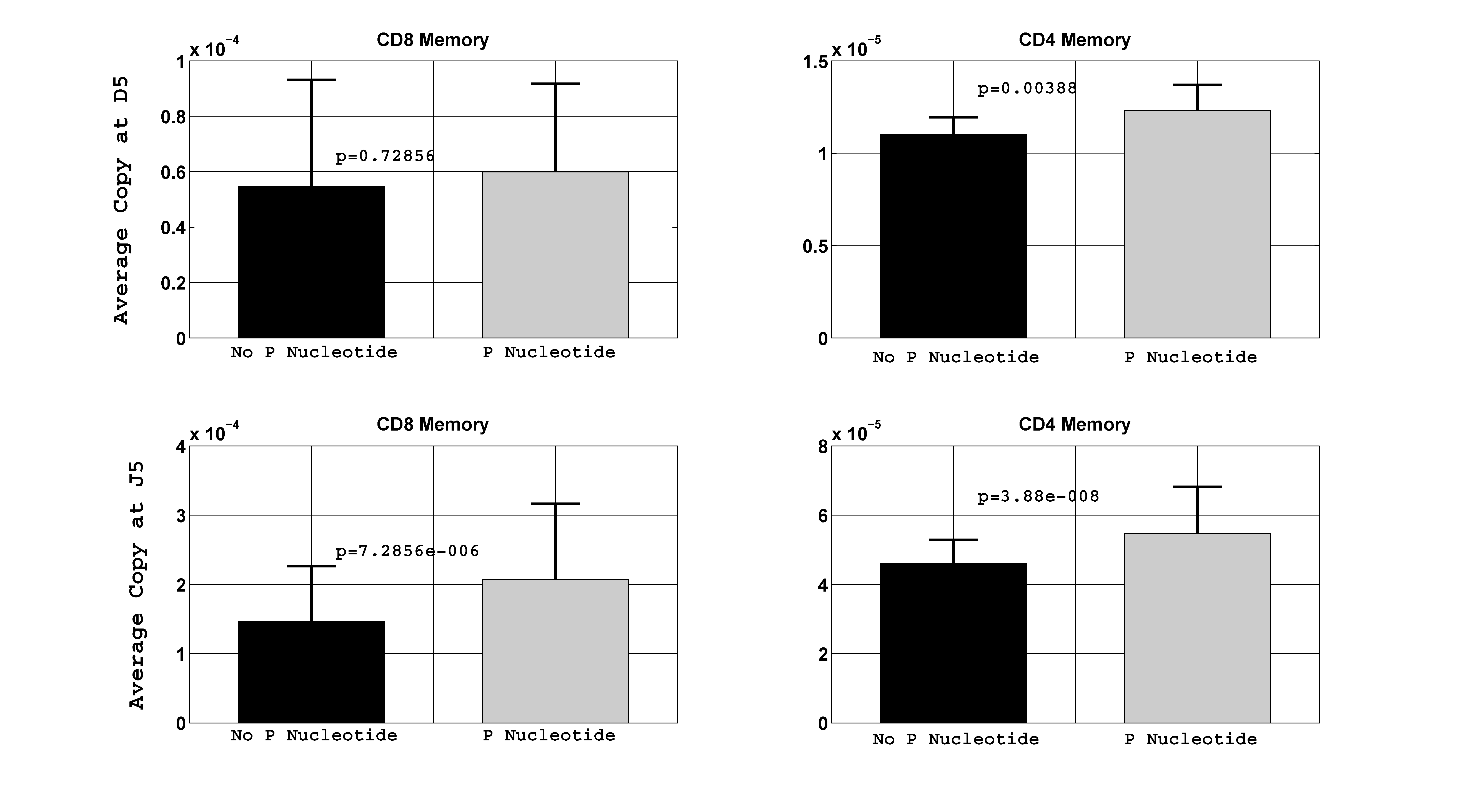

Supplement: Figure S1 — Average copy number with and without P nucleotide. Correlation between average copy number and P nucleotide at 5′ Dβ and 5′ Jβ gene segment in memory compartments of CD8+ and CD4+ T cells. Heights represent the mean of the sum of the normalized average copy number over Dβ gene subsets and similarly for Jβ gene segment subsets. Copy numbers were normalized by respective number of total reads in each donor. The error bar indicates one standard deviation. (TIF) [file pone.0052250.s001.tif]

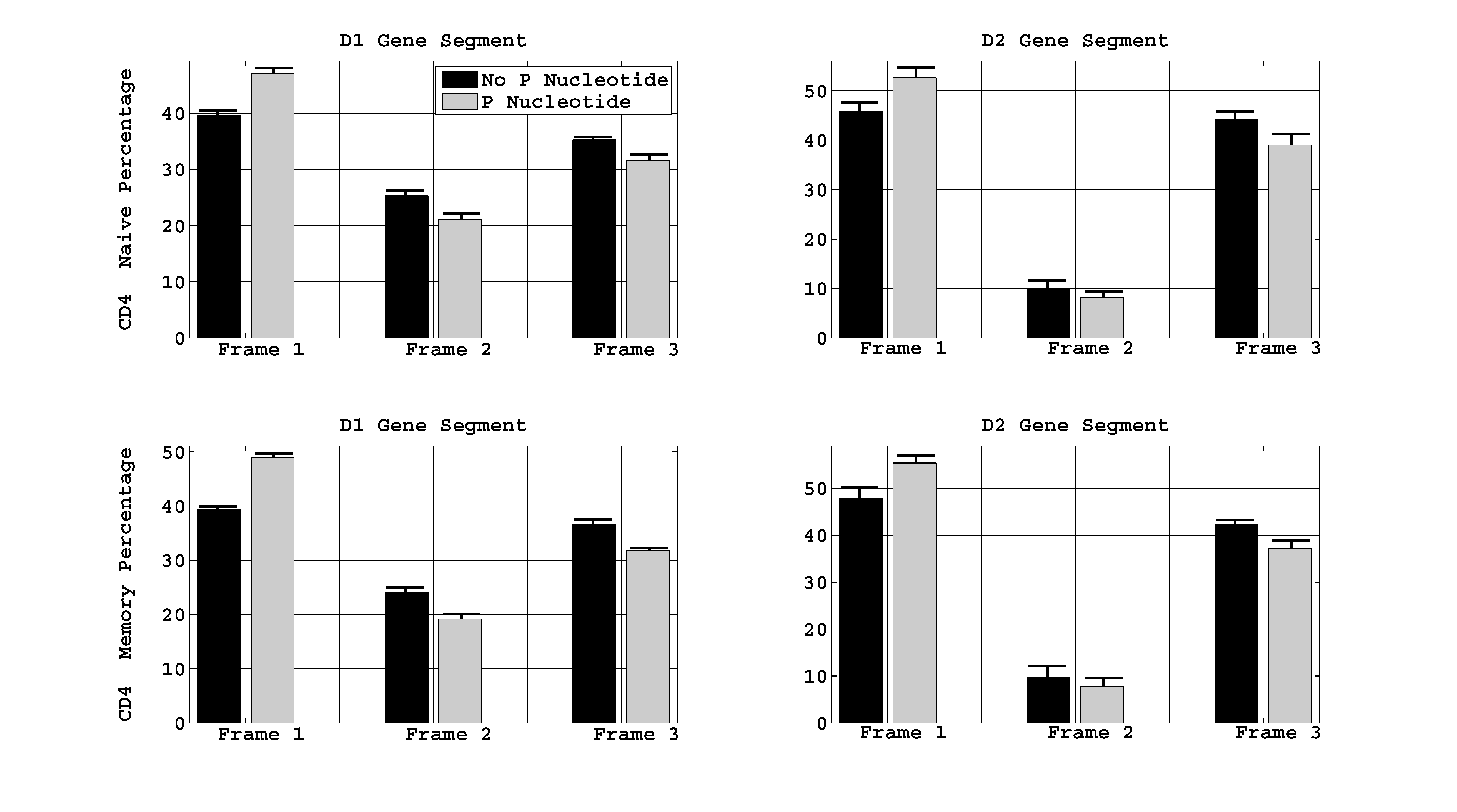

Supplement: Figure S3 — Reading frame biased induced by P nucleotide. Heights represent the mean percentage of sequence with a reading frame in P nucleotide and none P nucleotide. Results are shown for Dβ1 and Dβ2 gene segments in CD4+ naïve and memory T cells. Error bars indicate the standard deviation. (TIF) [file pone.0052250.s003.tif]
