# Supplementary material for: Palindromic Nucleotide Analysis in Human T Cell Receptor Rearrangements
Source: PLoS One. 2012 Dec 21;7(12):e52250. doi: 10.1371/journal.pone.0052250 (PMC3528771; doi:10.1371/journal.pone.0052250)
Supplement: Table S1 — Summary of CD8+ naïve and memory data set. Number of in-frame, TCRβ CDR3 nucleotide sequence, total reads and unique number of sequence obtained from the CD8+CD45RO−CD45RAhiCD62L+ (naïve) and CD8+CD45RO+CD45RAlow (memory) T-cell samples from each donor are given in the second and third columns respectively. The corresponding number of sequence with untrimmed coding ends at 3′Vβ, 5′Dβ, 3′Dβ, and 5′Jβ are shown in the last four columns. Each row shows the naïve (above) and memory (below) data. (DOC) [file pone.0052250.s004.doc]

| **Donor** | **Naïve-Memory Compartment** | | **Number of sequence with untrimmed coding ends** | | | | |
| --- | --- | --- | --- | --- | --- | --- | --- |
|  | Total Reads | Unique | 3’V | 5’D | 3’D | 5’J |  |
| **1** | 4,949,541  9,810,772 | 350,989  44,773 | 13,557  796 | 41,191  2,991 | 25,888  5,223 | 22,818  1,474 |  |
| **2** | 5,973,526  2,005,034 | 553,942  30,071 | 23,527  1,019 | 64,765  2,731 | 48,533  2,886 | 36,862  1,577 |  |
| **3** | 6,023,889  897,366 | 617,470  23,682 | 24,095  824 | 71,651  2,552 | 51,664  2,787 | 39,226  1,215 |  |
| **4** | 4,932,759  9,071,483 | 413,502  84,492 | 14,789  2,492 | 51,702  7,193 | 28,893  8,536 | 28,333  3,246 |  |
| **5** | 5,687,950  9,783,924 | 369,862  111,263 | 12,748  2,251 | 43,484  9,512 | 32,195  13,390 | 24,406  5,018 |  |
| **6** | 5,656,406  8,680,011 | 264,565  75,175 | 12,671  1,871 | 29,550  5,038 | 31,264  1,1331 | 17,575  3,105 |  |
| **7** | 5,759,173  9,361,795 | 216,263  55,107 | 9,552  1,028 | 26,887  3,422 | 22,729  6,307 | 16,061  1,875 |  |

|  |  | |  | | | | |
| --- | --- | --- | --- | --- | --- | --- | --- |
|  |  |  |  |  |  |  |  |
|  |  |  |  |  |  |  |  |
|  |  |  |  |  |  |  |  |
|  |  |  |  |  |  |  |  |
|  |  |  |  |  |  |  |  |
|  |  |  |  |  |  |  |  |
|  |  |  |  |  |  |  |  |

|  | | | | |
| --- | --- | --- | --- | --- |
|  |  |  |  |  |
|  |  |  |  |  |
|  |  |  |  |  |
|  |  |  |  |  |
|  |  |  |  |  |
