# Supplementary material for: Palindromic Nucleotide Analysis in Human T Cell Receptor Rearrangements
Source: PLoS One. 2012 Dec 21;7(12):e52250. doi: 10.1371/journal.pone.0052250 (PMC3528771; doi:10.1371/journal.pone.0052250)
Supplement: Table S2 — Summary of CD4+ naïve and memory data set. Number of in-frame, TCRβ CDR3 nucleotide sequence, total reads and unique number of sequence obtained from the CD4+CD45RO−CD45RAhiCD62L+ (naïve) and CD4+CD45RO+CD45RAlow (memory) T-cell samples from each of the six donors are given in second and third columns respectively. The corresponding number of sequence with untrimmed coding ends at 3′Vβ, 5′Dβ, 3′Dβ, and 5′Jβ are shown in the last four columns. Each row shows the naïve (above) and memory (below) data. (DOC) [file pone.0052250.s005.doc]

| **Donor** | **Naïve-Memory Compartment** | | **Number of sequence with untrimmed coding ends** | | | |
| --- | --- | --- | --- | --- | --- | --- |
|  | Total Reads | Unique | 3’V | 5’D | 3’D | 5’J |
| **1** | 7,372,437  7,949,975 | 320,483  182,259 | 13,429  7,885 | 38,052  20,964 | 38,626  22,499 | 23,989  12,531 |
| **2** | 7,545,658  8,191,562 | 367,817  158,813 | 13,321  4,821 | 46,057  17,116 | 27,866  12,506 | 25,763  9,540 |
| **3** | 7,748,954  7,652,009 | 376,564  200,923 | 15,012  7,706 | 46,056  23,371 | 36,903  20,403 | 26,579  13,030 |
| **4** | 8,767,076  9,552,123 | 363,716  168,344 | 12,161  5,529 | 41,932  18,175 | 40,028  19,300 | 23,814  9,932 |
| **5** | 8,562,368  8,105,040 | 250,072  155,043 | 10,048  6,068 | 31,050  18,322 | 24,713  16,251 | 18,407  10,652 |
| **6** | 8,731,451  8,584,644 | 361,725  172,136 | 15,048  7,024 | 43,451  19,774 | 35,865  18,292 | 26,296  11,957 |
