# Supplementary material for: Palindromic Nucleotide Analysis in Human T Cell Receptor Rearrangements
Source: PLoS One. 2012 Dec 21;7(12):e52250. doi: 10.1371/journal.pone.0052250 (PMC3528771; doi:10.1371/journal.pone.0052250)
Supplement: Table S3 — TdT probabilities estimate for all cell types at each of the four coding ends. Probability estimate of N nucleotide insertion is shown based on independent assumption. In order to avoid any potential bias middle regions of N2 and N1 segments are considered for the calculation of average nucleotide frequency, described in details in Methods Overview. (DOC) [file pone.0052250.s006.doc]

| **Mean estimates of TdT probabilities** | | | | |
| --- | --- | --- | --- | --- |
| Coding end | A | C | G | T |
| 3’V | 0.1540 | 0.2767 | 0.2516 | 0.3177 |
| 5’D | 0.2592 | 0.2915 | 0.2137 | 0.2356 |
| 3’D | 0.1817 | 0.2087 | 0.3967 | 0.2129 |
| 5’J | 0.2232 | 0.2535 | 0.2791 | 0.2441 |
