# Supplementary material for: Palindromic Nucleotide Analysis in Human T Cell Receptor Rearrangements
Source: PLoS One. 2012 Dec 21;7(12):e52250. doi: 10.1371/journal.pone.0052250 (PMC3528771; doi:10.1371/journal.pone.0052250)
Supplement: Table S4 — Demographic characteristic of palindromic sequences. In each donor the fraction of palindromic inserts of up to six bases long is shown against the coding ends in CD8+ naïve T cells. The 5′Jβ does not show any variation while the 3′Vβ shows some fluctuations with regard to the HLA. The two related siblings, donor 1 and donor 3, show the similar pattern of palindromes at 3′Vβ, 5′Dβ and 5′Jβ coding ends. (DOC) [file pone.0052250.s007.doc]

| **Donor** | **Age/Sex/**  **Geographic origin** | **HLA-A** | **HLA-B** | **HLA-C** | **3’V** | **5’D** | **3’D** | **5’J** |
| --- | --- | --- | --- | --- | --- | --- | --- | --- |
| 1 | 25 F  Caucasian | *0201/*0101 | *44/*57 | *06/*16 | 0.028 | 0.060 | 0.020 | 0.027 |
| 2 | 51 F  Caucasian | *01/*24 | *38/*57 | *06/*12 | 0.033 | 0.060 | 0.028 | 0.027 |
| 3 | 22 F  Caucasian | *0201/*0101 | *44/*57 | *06/*16 | 0.029 | 0.059 | 0.026 | 0.026 |
| 4 | 39 M  Japanese | *26/*31 | *07/*56 | *01/*07 | 0.027 | 0.065 | 0.019 | 0.028 |
| 5 | 38 M  Indian | *0205/*24 | *35/*35 | *04/*12 | 0.025 | 0.060 | 0.027 | 0.027 |
| 6 | 20 F  African | *01/*34 | *0801/*53 | *04/*07 | 0.036 | 0.056 | 0.043 | 0.026 |
| 7 | 53 M  African | *23/*33 | *57/*58 | *10/*18 | 0.031 | 0.063 | 0.033 | 0.029 |
